# Sensitivity Analysis of a Mathematical Model Simulating the Post-Hepatectomy Hemodynamics Response

**DOI:** 10.1007/s10439-022-03098-6

**Published:** 2022-11-03

**Authors:** Lorenzo Sala, Nicolas Golse, Alexandre Joosten, Eric Vibert, Irene Vignon-Clementel

**Affiliations:** 1 Inria Saclay Ile-de-France, 91120 Palaiseau, France; 2 Université Paris-Saclay, Inserm Physiopathogénèse et traitement des maladie du foie, UMR-S 1193, 94800 Villejuif, France

**Keywords:** Hepatectomy, Lumped parameter mathematical model, Virtual patients, Sensitivity analysis, Sobol indices, Polynomial chaos expansion method

## Abstract

Recently a lumped-parameter model of the cardiovascular system was proposed to simulate the hemodynamics response to partial hepatectomy and evaluate the risk of portal hypertension (PHT) due to this surgery. Model parameters are tuned based on each patient data. This work focuses on a global sensitivity analysis (SA) study of such model to better understand the main drivers of the clinical outputs of interest. The analysis suggests which parameters should be considered patient-specific and which can be assumed constant without losing in accuracy in the predictions. While performing the SA, model outputs need to be constrained to physiological ranges. An innovative approach exploits the features of the polynomial chaos expansion method to reduce the overall computational cost. The computed results give new insights on how to improve the calibration of some model parameters. Moreover the final parameter distributions enable the creation of a virtual population available for future works. Although this work is focused on partial hepatectomy, the pipeline can be applied to other cardiovascular hemodynamics models to gain insights for patient-specific parameterization and to define a physiologically relevant virtual population.

## Introduction

Liver surgery is one of the only curative treatments for primary or secondary liver tumors. Percutaneous ablation is a less invasive alternative but cannot be proposed for all tumors, while liver transplantation is reserved only for hepatocellular carcinomas (HCC, the most frequent primary tumors), under strict conditions related to the tumor extend and the patient condition. Hepatic resection, indicated in the absence of extrahepatic tumor extension, therefore allows for tumor removal and lymph node dissection. However, the main risk of hepatectomy is the occurrence of post-hepatectomy liver failure (PHLF), the incidence of which depends on the extent of the resection, the quality of the underlying parenchyma, the parameters related to the operation, the patient’s characteristics and the postoperative complications.^19^ Despite the fact that the liver has the ability to regenerate after a large tissue loss, several patients suffer from the *small-for-size syndrome*,^20^ which leads to PHLF and, sometimes, to death. The EASL (European Association for the Study of the Liver) recommendations are specifically designed for HCC but can be extrapolated to other types of cancer.^10^ They recommend performing the surgery based on functional scores (Child–Pugh and Model for End-stage Liver Disease^17^), on the pre-operative portal hypertension (PHT) condition and on the liver resection extent. If preoperative PHT is clearly a major predictor of PHLF,^6^ in literature, however, some papers have shown that post-operative PHT may also be a major trigger for PHLF.^1^ Yet currently there is no method to predict such hemodynamics response to the surgery. Our group^12^ proposed a lumped-parameter mathematical model (“Appendix 1”), which—starting from preoperative clinical/radiological and pre-resectional hemodynamic data—predicts the risk of post-resectional PHT by simulating the hemodynamics changes due to partial hepatectomy in the entire circulation. The use of such simplified model to simulate the cardiovascular system exploiting the electric analogy to fluid flow and represent organs as compartments is a trade-off between the accuracy needed in the clinics for this type of surgery and the complexity that such models may require to fully describe the human body hemodynamics as already presented in the literature (e.g. Refs. 5, 15).

The present work stems from the interest on extending the analysis of the hemodynamics model proposed in Ref. 12 that has proved to be clinically relevant. In particular the ambition of the current research is twofold: (i) perform a sensitivity analysis (SA) study to identify the most significant model parameters (inputs) with respect to the main postoperative clinical outputs of interest, and (ii) create a virtual population representative of a real patient cohort available for future studies. Beyond these goals, the current study examines also the possibility to better use the clinical resources in the parameter calibration process by fixing the inputs that have negligible effect on the selected outputs and by increasing the preoperative clinical measurement accuracy needed to estimate the significant model inputs. New insight emerges from the stochastic parameter sampling of the deterministic model presented in Ref. 12.

The use of a SA methodology to investigate the influence of inputs (“Input Parameters” section) to clinically relevant quantities of interest (“Quantities of Interest” section) is fundamental due to the presence of several organ compartments and nonlinear elements, which makes the interactions among parameters and outputs non trivial. Moreover, the physiological response to the surgery is not trivial to anticipate due to the double—arterial and venous—perfusion of the liver and the closed-loop nature of the blood circulation. The use of digital twins combined with SA allows to isolate the effect of single parameters, which cannot be directly assessed with patient data.

In the literature some SA works included open-loop models, e.g. Refs. 14, 25, whereas very few involved a closed-loop systems. Ellwein *et al*.^9^ highlighted the importance of performing SA for such system but with a local SA. Here the aim is to take into account the variability within a population, namely the range of patients undergoing partial hepatectomy. Marquis *et al*.^16^ used structured correlation method in the context of local SA to determine parameters identifiability for a closed-loop cardiovascular model exploiting also experimental data. In Ref. 4 the authors conducted a synthetic-data-based parametric study for a closed-loop cardiovascular system in order to investigate sensitive and insensitive model parameters trying to decrease the complexity of the model. As reviewed in Ref. 8, global sensitivity analysis (GSA) for cardiovascular models has already shown its usefulness and, when combined with the polynomial chaos expansion (PCE) method, its efficiency. Wang *et al*.^24^ showed the potential of a lumped-parameter model combined with GSA to understand the drivers of the mismatch between the gold-standard portocaval pressure gradient (PCG) measurement and the hepatic-venous portal gradient, a surrogate measurement. The accuracy of the latter is important for the diagnosis of PHT. The model consisted of a very detailed closed-loop system with a refined description of the liver structure. The GSA adapted by the authors was a Sobol index analysis that took into account the variance of six resistances, focusing on the liver and liver-feeding splanchnic system. These variances were set to reflect the correlation between these two measurements in this population. Moreover, Refs. 14, 25 provide and discuss the generation of virtual patient cohorts in the context of one-dimensional hemodynamics modeling with selection criteria; in the current work the generation of a virtual patient database with a similar methodology is complemented with a novel comparison not only with literature data but also with measurements. Finally, we refer to Ref. 21 for a more detailed recent review of SA methods applied in this context. The main SA novelties that this paper is bringing are briefly introduced in the next paragraph and in particular in “Classical Polynomial Chaos Expansion” section.

This study is based on a lumped parameter model^2,12^ briefly recalled in the Method section. That section also presents the chosen GSA method based on Sobol indices and the PCE approach that will later be used. The input parameter distributions are computed from patient data. Thus, the considered ranges are by design reflecting the variability in the population: this is a strength of the analysis, by contrast to other GSA hemodynamics papers where parameter ranges are often chosen ad-hoc. A first GSA highlights the need for a physiological filter, which to our knowledge is not discussed in the literature. We then propose an efficient approach based on PCE to perform GSA based on the already computed simulations. As a last contribution, the SA results are presented and their consequences for a better parameter estimation strategy discussed.

## Model and Methods

This section first introduces the model and the associate inputs and outputs, then the SA methodologies employed in this work, namely the Sobol indices, the classical PCE strategy and a novel PCE-based method.

### Human Cardiovascular Lumped-Parameter Model

**Figure 1:**
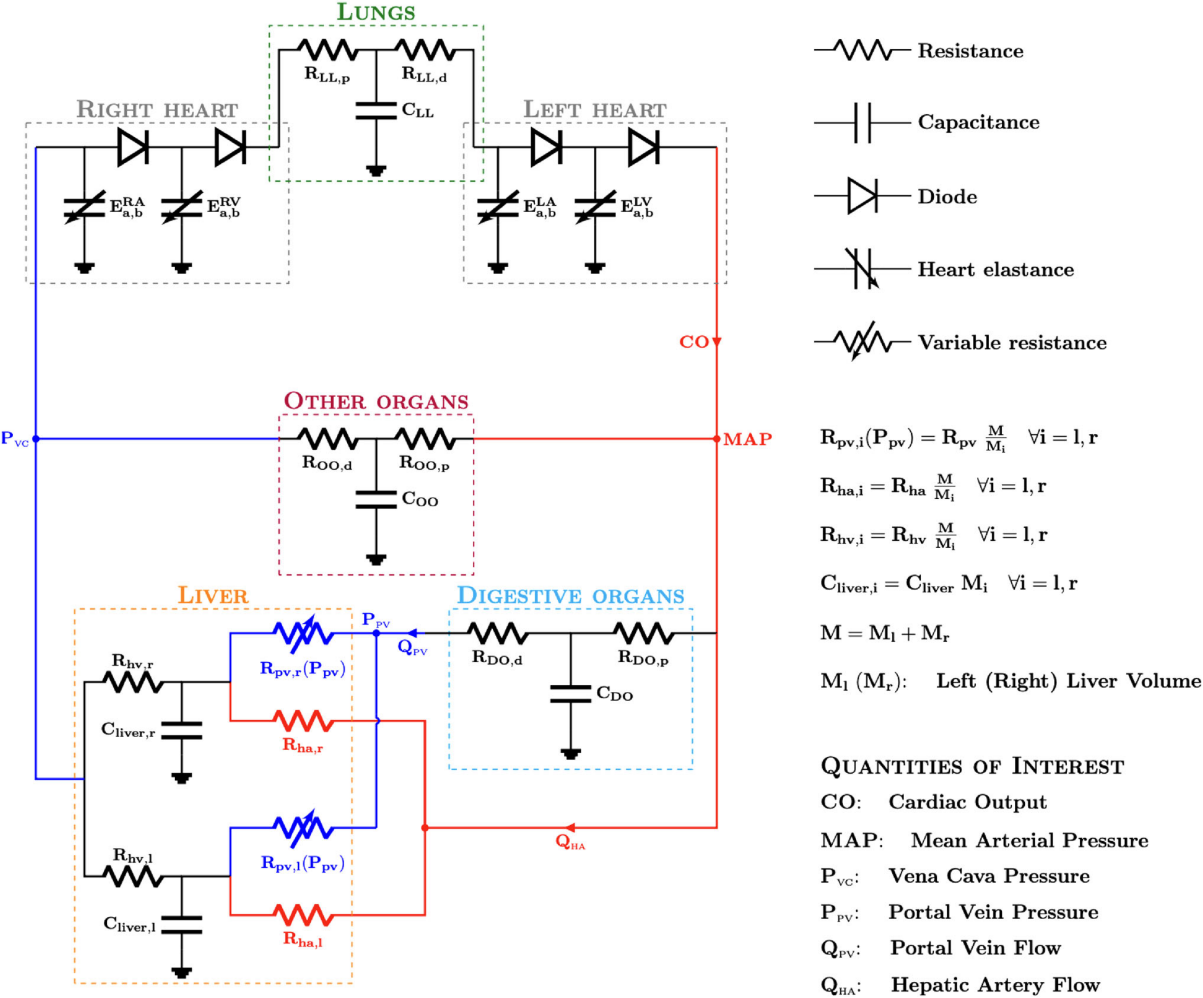
Lumped-parameter model representing the human cardiovascular system with a zoom on liver. Note that besides the quantities of interest already annotated in the diagram, the portocaval gradient PCG is the difference between $P_{\text {pv}}$ and $P_{\text {vc}}$ (see “Quantities of Interest” section). Originally developed in Refs. 2, 12.

The lumped-parameter model utilized in this work has been already presented in Refs. 2, 12, and it represents the human cardiovascular system simulating the hemodynamics response to partial hepatectomy (Fig. 1). “Appendix 1” recalls the main features of such model. Hereafter we refer to this model as the *full model* ${\mathcal {M}}$ described by the following equation:

1 $$ Y = {\mathcal {M}} (X), $$

where *X* and *Y* represent the input and output vectors, respectively.

#### Input Parameters

The following parameters are considered as input vector *X* for the SA study proposed in this work:

- heart elastances with the right atrium and left ventricle ($E_{{\text {a}},{\text {RA}}}$, $E_{{\text {b}},{\text {RA}}}$, $E_{{\text {a}},{\text {LV}}}$, $E_{{\text {b}},{\text {LV}}}$);
- resistances to the flow within the PV, HA, hepatic vein, total digestive organs, and other organs ($R_{\text {pv}}$, $R_{\text {ha}}$, $R_{\text {hv}}$, $R_{\text {DO}}$ and $R_{\text {OO}}$, respectively);
- fraction of the total liver mass to be resected during the surgery (Hpx); in this case to simplify and have a consistent analysis throughout the sampling, the resected mass is first subtracted from the right liver, then when necessary from the left part.

The selection of this subset of model parameters is driven on one hand by the high computational cost that a robust and accurate (see “Results” section) Sobol indices analysis on all parameters would have required, and on the other hand the study is driven by the outcome of Ref. 12. The distributions of such parameters, indeed, are directly derived from Ref. 12, in particular from their patient cohort data (more details in “Appendix 2”).

**Figure 2:**
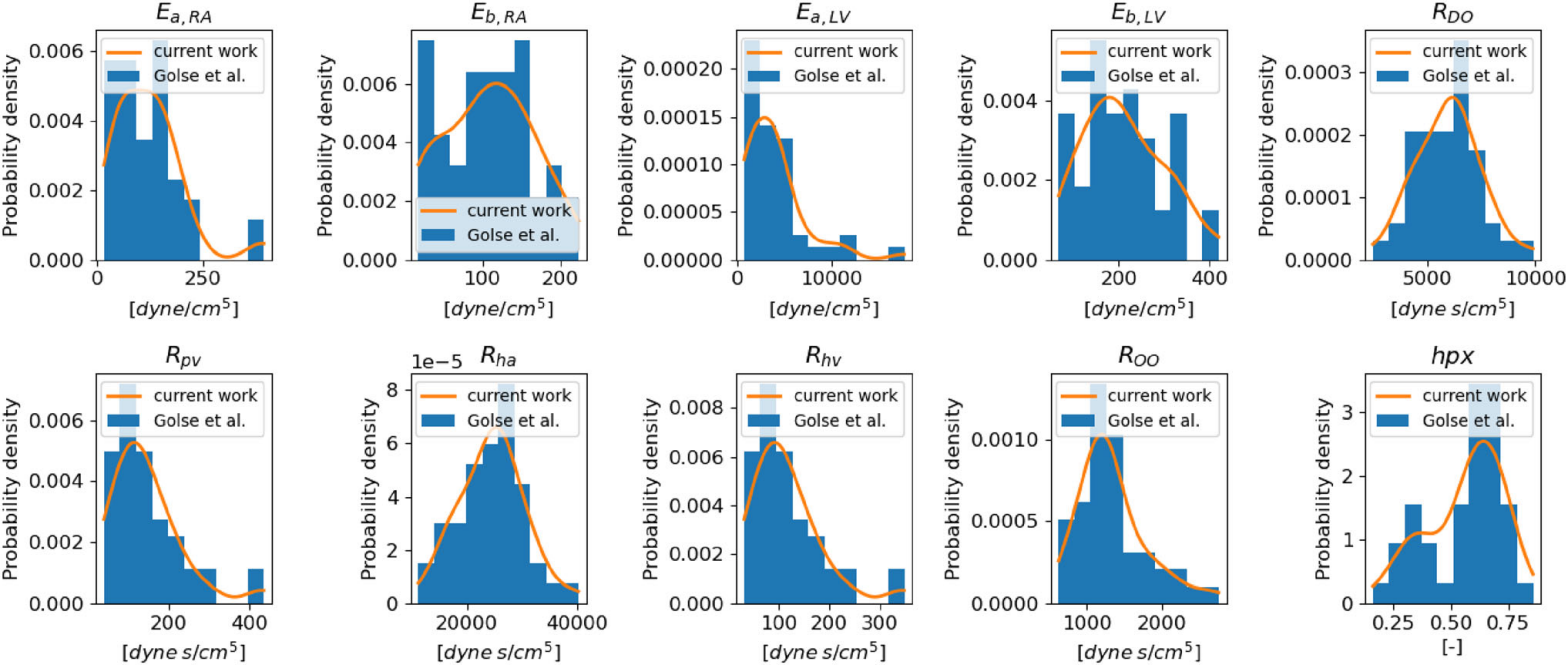
Comparison between the probability density distribution of the patient cohort of input parameters employed by Golse *et al*.^12^ (blue) and the associated estimated empirical distribution computed *via* the kernel density estimation (orange). Recall that hpx is the fraction of the total liver mass to be resected during the surgery.

GSA requires probability density functions in order to perform the needed input sampling. Thus, empirical distributions are computed, *via* the kernel density estimation. They regularize the original dataset distributions (Fig. 2). The best fit known distribution reported for each input parameter in Table 1 varies among parameters. Eventually, for the results presented in this work in “Results” section, the prior distributions of the SA study are the empirical ones as they are naturally bounded to the range provided by the data. Moreover, since the data used are from a real population cohort with one set of measurements per individual, this study limits its investigation only to the variability between different subjects, rather than the parameters variability within the same patient.

**Table 1:** Best fit known distribution for the input parameters selected for the SA study.

Input	$E_{{\text {a}},{\text {RA}}}$	$E_{{\text {b}},{\text {RA}}}$	$E_{{\text {a}},{\text {LV}}}$	$E_{{\text {b}},{\text {LV}}}$	$R_{\text {DO}}$	$R_{\text {pv}}$	$R_{\text {ha}}$	$R_{\text {hv}}$	$R_{\text {OO}}$	Hpx
Best fit	*Logistic*	*Uniform*	*Exponential*	*Normal*	*Logistic*	*Cauchy*	*Logistic*	*Cauchy*	*Cauchy*	*Cauchy*

#### Quantities of Interest

In this part, the quantities of interest for this study are listed among all the possible outputs that the model ${\mathcal {M}}$ is able to provide. With a slight abuse of notation, we denote with *Y* the vector representing these quantities of interest. More precisely, these selected outputs *Y* are computed as the mean value over a cardiac cycle at the beginning and end of the surgery—pre-hpx and post-hpx, respectively. See “Appendix 3” for more details on how these scalar quantities are computed from the time-dependent variables.

Motivated by the clinical needs to evaluate the patient state during hepatectomy, the quantities of interest annotated also in Fig. 1 for the SA study are:

- PV pressure $$P_{\text {pv}}$$;
- portocaval gradient PCG, which is the pressure difference between the PV and the inferior vena cava;
- systemic arterial pressure, called MAP in the clinics;
- cardiac output CO;
- blood flow in the HA ($Q_{\text {ha}}$) and in the PV ($Q_{\text {pv}}$).

All these quantities can be eventually measured in specific moments during the surgery as described in Ref. 12.

### Sensitivity Analysis

SA is *the study of how uncertainty in the output of a model (numerical or otherwise) can be apportioned to different sources of uncertainty in the model input*.^22^ In literature a multitude of different methods is provided to perform SA. This variety is due to the fact that SA is employed with various goals: e.g. model verification and understanding, model simplifying and factor prioritization, aid in the validation of a computer code, guidance research effort, and justification in terms of system design safety.^13^

#### Sobol Indices

This work focuses on Sobol indices, a variance decomposition-based method, which expresses the share of variance of an output that is due to a given input or input combination. The motivations behind the choice of this approach in order to reach our goal are the global exploration in the space of the model input parameters, and the property of being a non-intrusive method with respect to the analyzed mathematical model.

Therefore, let the input parameters $\left\{ X_{j} \right\} _{j \in \left[ 1, \dots , d \right] }$ be random independent variables following each a probability distribution, employed to compute the random output vector *Y* *via* the model ${\mathcal {M}}$. The first and the total Sobol order indices are then respectively defined as:

2a $$ S_{{ij}}  = \frac{{\text{var} [{\mathbb{E}}(Y_{i} |X_{j} )]}}{{\text{var} [Y_{i} ]}}\quad \forall i \in [1. \ldots N_{{{\text{outputs}}}} ], $$

2b $$ S_{{ij}}^{{{\text{tot}}}}  = 1 - \frac{{\text{var} [{\mathbb{E}}(Y_{i} |X_{{ - j}} )]}}{{\text{var} [Y_{i} ]}} = 1 - S_{{( - ij)}} \quad \forall i \in [1. \ldots N_{{{\text{outputs}}}} ] $$

In Eq. (2) ${{\,{\text{var}}\,}}$ denotes the variance, ${\mathbb {E}}$ the expected value, and $X_{(-j)} = \left( X_{1}, \dots , X_{j-1}, X_{j+1}, \dots , X_{d} \right)$. The interpretation of Eq. (2a) can be read as follows: ${{\,{\text{var}}\,}}[Y_{i}]$ corresponds to the overall variability of $Y_{i}$ including nonlinear effects, while ${{\,{\text{var}}\,}}[{\mathbb {E}}[Y_{i}|X_{j}]]$, the variance of conditional expectation ${\mathbb {E}}(Y_{i}|X_{j})$, represents the main or first order effect of $X_{j}$ on $Y_{i}$. For instance, if $Y_{i}$ is sensitive to $X_{j}$, then ${\mathbb {E}}(Y_{i}|X_{j})$ is likely to vary a lot implying a high value of ${{\,{\text{var}}\,}}[{\mathbb {E}}(Y_{i}|X_{j})]$, thus the value of $S_{ij}$ is close to 1. The total order Sobol index in Eq. (2b), on the other hand, describes the influence of the specific input $X_{j}$ and its interaction with the other inputs $X_{(-j)}$ with respect to the output $Y_{i}$. If the value of the total order Sobol index $S^{\text {tot}}_{ij}$ is close to 0, then the output $Y_{i}$ is considered insensitive to the input $X_{j}$. High order interactions can also be evaluated with high order Sobol indices, see Ref. 18. We remark that recently in literature time-dependent Sobol indices have been proposed,^7^ however given the quantities of interest needed from a clinical viewpoint, this work does not require such novelties at this stage.

To numerically compute the sensitivity indices described in Eq. (2) several approaches have been proposed in literature.^18^ In the current study, the so-called Saltelli algorithm has been adopted, which is a quasi-Monte Carlo approach that exploits the estimation proposed in Ref. 22 on the basis of a combinatoric argument. The computational cost of such estimator for first and total order Sobol indices is $N_{\text {s}} = (d+2) \, N$ model evaluations where *d* is the dimension of the input space *X* and *N* is the sample size.

From a theoretical viewpoint, the computational cost required by the number of model evaluations in this approach can still be very high, depending on the computational cost of a single model evaluation. In literature, several numerical techniques have been proposed to reduce the overall computational cost. The main idea is to build a reduced model—called metamodel or surrogate model—which is able to reproduce with a specified accuracy the behavior of the full model at a lower computational cost. Usually this reduced model is based on polynomials, splines, generalized linear models, Gaussian processes and many other possibilities, which rely on different hypotheses. A very common approach is the PCE method.^8^

#### Classical Polynomial Chaos Expansion

The classical PCE technique is a well-known uncertainty quantification spectral method used to substitute the dynamics of an expensive-to-compute numerical model (in this work it corresponds to the full model ${\mathcal {M}}$), with an inexpensive-to-compute metamodel, denoted hereafter with ${\mathcal {M}}^{\text {PCE}}$, representing the output of the model by a polynomial function of its inputs [from Eq. (1)]:

$$\begin{aligned}&Y = {\mathcal {M}}(X) = \sum _{k=0}^{\infty } \beta _{k} \varPsi _{k} (X), \\&\Rightarrow Y \approx {\mathcal {M}}^{\text {PCE}} (X) = \sum _{k=0}^{P} \beta _{k} \varPsi _{k} (X), \end{aligned}$$

where $P+1$ is the number of PCE coefficients $\beta _{k}$, $\varPsi _{k} (X)$ is the *d*-dimensional polynomial basis that is orthonormal with respect to the joint probability density function of the input *X*. In this study $P=\dfrac{(d + q)!}{d!\,q!}$ where $q=4$ is the maximum degree of the polynomial basis. The coefficient vector $\beta = [ \beta _{k} ]_{k=0}^{P}$ can be calculated adopting the least-square method as follows:

$$\begin{aligned}&Y = {\mathcal {M}} (X) = \sum _{k=0}^{P} \beta _{k} \varPsi _{k}(X) + \varepsilon _P \Rightarrow \beta ^{\text {T}} \varPsi (X) \approx {\mathcal {M}}(X) \\&\Rightarrow \beta ^{*} = {\text {argmin}}_\beta {\mathbb {E}} \left[ \left( \beta ^{\text {T}} \varPsi (X^{(N_{\text {s}})}) - {\mathcal {M}}(X^{(N_{\text {s}})}) \right) ^{2} \right] , \end{aligned}$$

where $X^{(N_{\text {s}})}$ are the input parameters values derived from $N_{s}$ full model ${\mathcal {M}}$ evaluations. Note that due the orthonormality of the surrogate PCE model, the model variances (partials and total) can be calculated only using the expansion coefficients $\beta _{k}$, thus the Sobol indices are computed *for free*. The following error estimation based on the predictive squared correlation coefficient $Q^{2}$ evaluates the PCE accuracy:

3 $$ Q^{2} = 1 - \dfrac{\sum _{l=1}^{N_{\text {test}}} \left( Y^{(l)} - {\mathcal {M}}^{\text {PCE}}(X^{(l)} \right) ^{2}}{N_{\text {test}} \, {{\,{\text{var}}\,}}(Y)}, $$

where $$Y^{(l)} = {\mathcal {M}}(X^{(l)})\, \forall l = 1, \dots , N_{\text {test}}$$ and $$\left\{ X^{(l)} \right\} _{l = 1, \dots , N_{\text {test}}} \cap \left\{ X^{(k)} \right\} _{k = 1, \dots ,N_{\text {s}}} = \emptyset $$.

#### Physiologically Filtered PCE

In this section the methodological novelty of this work is proposed. The input–output framework described in “Human Cardiovascular Lumped-Parameter Model” section is not guaranteeing that all the considered outputs *Y* have physiological values. Thus, a physiological filter is applied to the pre-hpx results computed with the full model ${\mathcal {M}}$. The output selection ranges reported in Table 2 have been defined by the expertise of the team (co-authors that are anesthesiologist and surgeons at a major liver surgery center in France) and literature.^6,10^

**Table 2:** Physiological filter applied on the pre-hpx outputs following the clinical advice

Clinical output	Minimum	Maximum	Units
MAP	50	130	mmHg
CO	3	10	L/min
$P_{\text {pv}}$	3	20	mmHg
PCG	1	14	mmHg

The filtering consists in discarding simulations outside of these ranges, thus removing the corresponding input–output couples. After the filtering, from a classical Sobol experiment the number of remaining filtered physiological simulations can be significantly decreased. If this reduction occurs, the span of the parameter space is non-balanced and the hypothesis to apply the Saltelli algorithm is not valid anymore. Thus, an innovative strategy exploiting the PCE method is proposed.

The idea of this approach is to use only the filtered input–output couples—for the notation decreased from size *N* to size $N^{*}$—to build the PCE that would represent the physiological surrogate model ${\mathcal {M}}^{\text {PCE}}$ of our full model ${\mathcal {M}}$. In particular, the orthonormal basis of the PCE is built using only such couples employing the adaptive Stieltjes algorithm,^23^ a more stable alternative to the well-known Gram–Schmidt algorithm. The coefficients of this PCE-based surrogate model are then used to compute analytically the novel Sobol indices.

In the current work the GSA including the computation of Sobol indices and the construction of the PCE have been performed in Python with the library Openturns.^3^

## Results

First, this section introduces the GSA performed by solving the full model ${\mathcal {M}}$ to investigate the influence of the input parameters *X* on the quantities of interest *Y* (see “Human Cardiovascular Lumped-Parameter Model” section). Second, the physiological filter with the novel PCE-based methodology is applied and the associated results in terms of GSA and virtual population are presented. Finally, the outcomes of a preliminary study on improving the calibration step for the model ${\mathcal {M}}$ are exhibited.

### Sensitivity Analysis Results Using the Full Model

**Figure 3:**
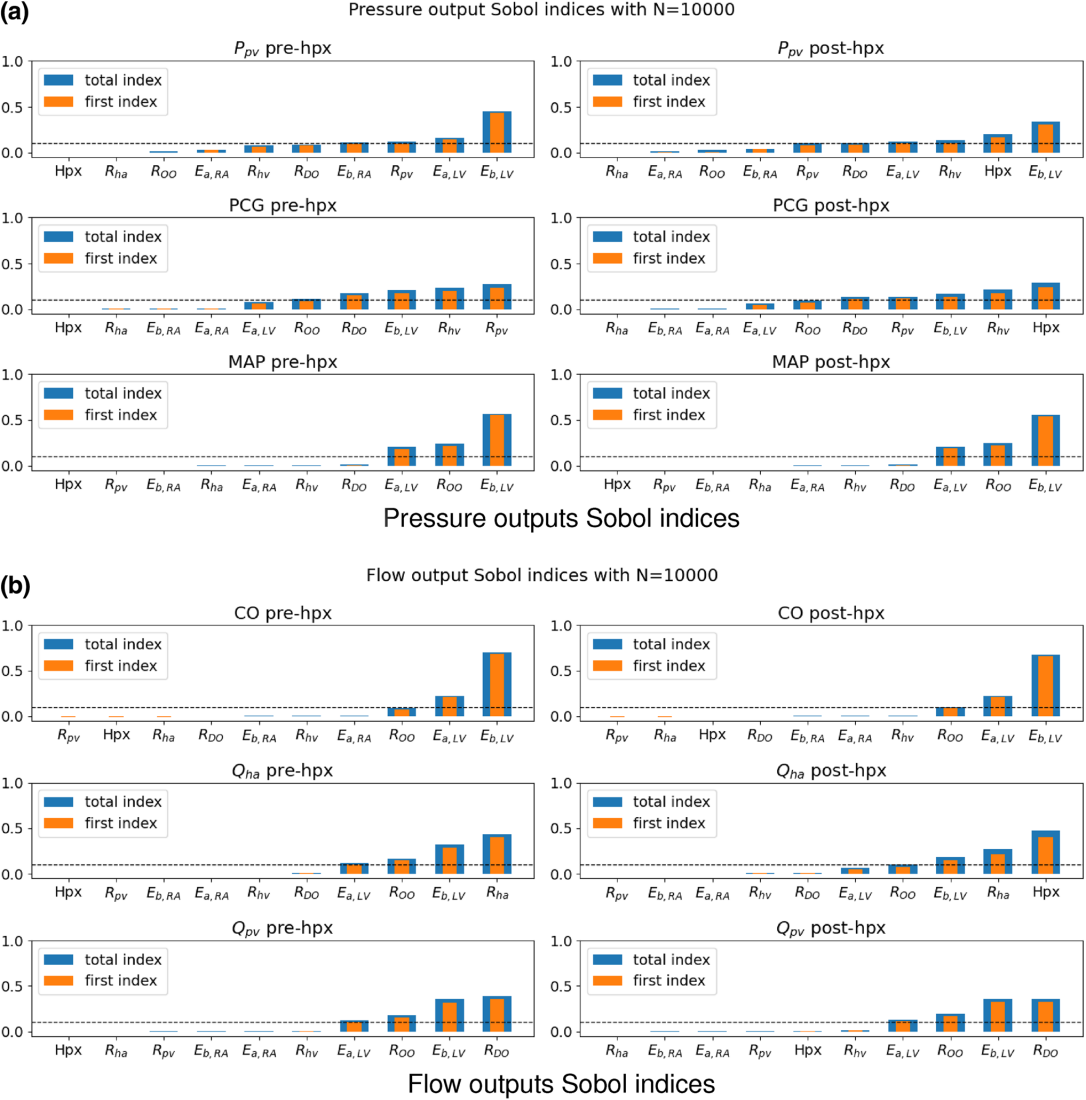
SA results using the full model ${\mathcal {M}}$ before (pre-hpx) and after (post-hpx) the virtual hepatectomy ($N=10^{4}.$)

The Sobol indices analysis using the Saltelli algorithm (see “Sobol Indices” section) is performed applying the previous empirical distributions shown in Fig. 2 with $N=10^{4}$, thus $N_{\text {s}} = 10^{4} \, (d+2)$ with $d=10$. The choice of *N* has been optimized in terms of accuracy–efficiency performing multiple SA experiments similarly to what is reported later on in Tables 5 and 6. The results of the GSA are illustrated in Fig. 3.

Figure 3 suggests that $E_{{\text {a}},{\text {LV}}}$  and $E_{{\text {b}},{\text {LV}}}$  play a significant role in all the hemodynamics quantities of interest, both before and after the virtual hepatectomy. In particular, the couple of heart elastances in the left ventricle combined with the other organ resistance $R_{\text {OO}}$ have the largest impact on the driving force of the cardiovascular system (MAP and CO, pre-hpx and post-hpx). The model predictions suggest that the HA resistance ($R_{\text {ha}}$) is significantly influencing the value of the HA flow—as expected–whereas it has a negligible effect on all the other outputs. Moreover, the Sobol indices point out that the quality of the estimation of $R_{\text {DO}}$ affects remarkably $Q_{\text {pv}}$, but mildly $P_{\text {pv}}$ and PCG. Finally, regarding $R_{\text {pv}}$ and $R_{\text {hv}}$, Fig. 3a shows that they are key factors only in the determination of the predicted $P_{\text {pv}}$ and PCG. The main difference between pre-hpx (left panels Fig. 3) and post-hpx (right panels Fig. 3) is the great impact of the size of the virtual hepatectomy (Hpx) for the post-hpx predictions of $P_{\text {pv}}$, PCG and $Q_{\text {ha}}$. Due to its strong role in the post-hpx, the other input variables that were playing role in the pre-hpx phase have a reduced effect on the outputs mentioned above. In line with Refs. 2, 12, Hpx has a negligible effect on the post-hpx $Q_{\text {pv}}$, MAP and CO in comparison with the main driving parameters of the systemic blood circulation ($E_{{\text {a}},{\text {LV}}}$, $E_{{\text {b}},{\text {LV}}}$  and $R_{\text {OO}}$). A summary of these results, denoting the sensitive—when $S_{ij} \gg 0.1$—and insensitive parameters—when $S^{\text {tot}}_{ij} \approx 0$—for each clinical output is displayed in Table 3.

**Table 3:**
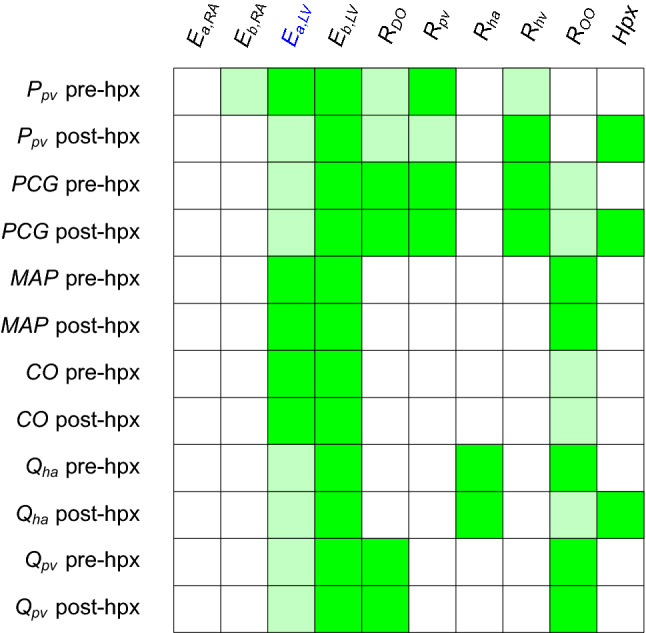
Sensitive and insensitive input parameters for each quantity of interest suggested by the GSA performed with the full model ${\mathcal {M}}$ (see Fig. 3 for detailed representation of Sobol indices).

Legend: green = sensitive $S_{ij} \gg 0.1$, light green = fairly sensitive $S_{ij} \approx S^{\text {tot}}_{ij} \approx 0.1$, white = insensitive $S^{\text {tot}}_{ij} \approx 0$

**Figure 4:**
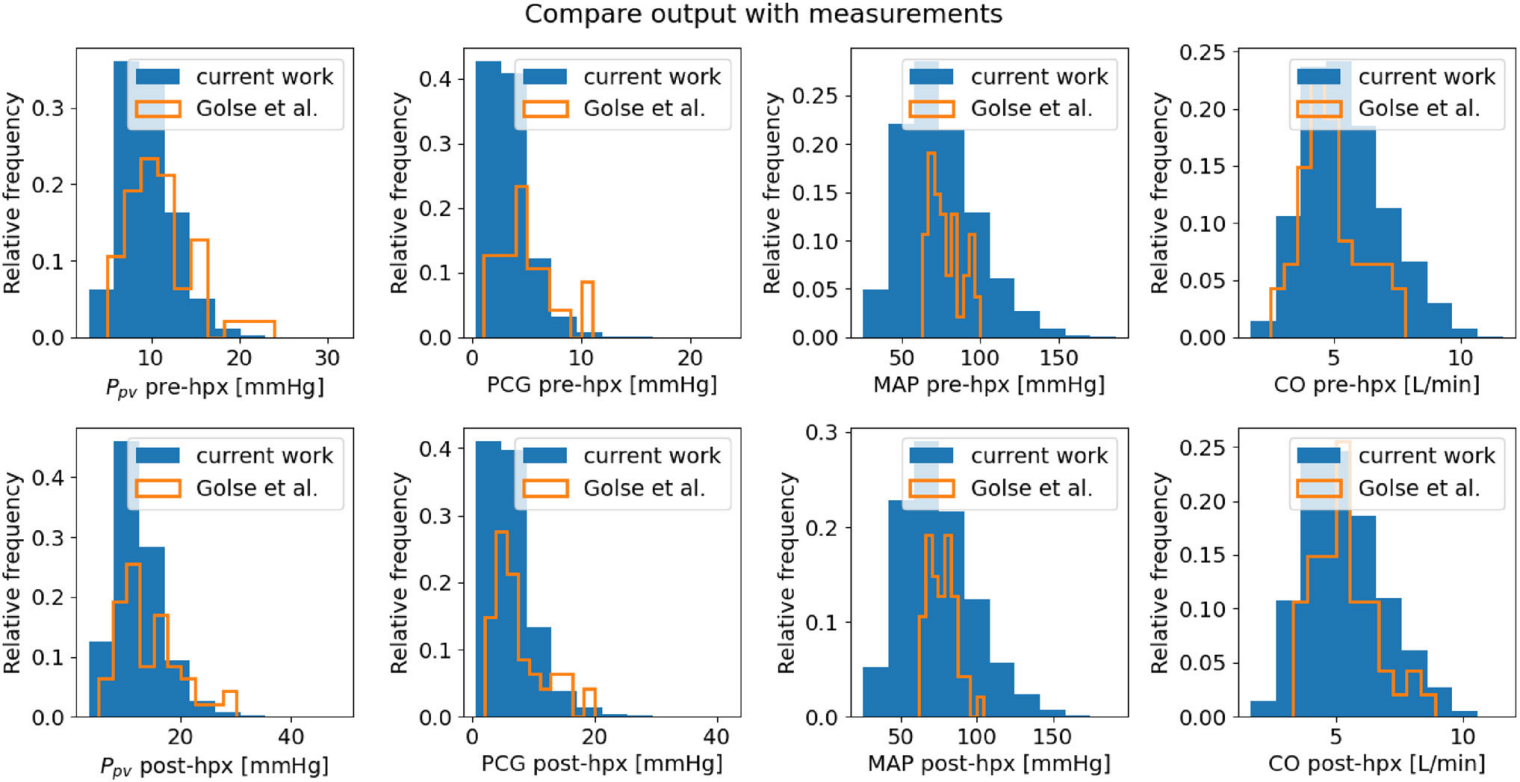
Output probability density function comparison between clinical measurements from Golse *et al*.^12^ (orange) and full model ${\mathcal {M}}$ simulation results with $N=10^{4}$, thus $N_{\text {s}} = 1.2\times 10^{5}$ (blue). The *y*-axis displays the relative frequency, which is the ratio of the frequency of a particular event to the total frequency of that event to happen.

**Comparison with clinical measurements.** Figure 4 displays the predicted probability density functions for the major hemodynamics outputs *Y* and compares them with the associated clinical measurement distributions from Ref. 12.

These results show that there is overall a good agreement between the simulation predictions and the measures, especially for $P_{\text {pv}}$ and PCG both pre-hpx and post-hpx, which are the two main assessed factors to evaluate the practicability and the success of this type of surgery. Using as baseline value the median of the clinical measurements from Ref. 12, we compute the difference with respect to the median of the distribution of our simulation results. For pre-hpx and post-hpx $P_{\text {pv}}$ the difference is below 1 mmHg ($8\%$) and between 1 and 1.6 mmHg ($\sim 25\%)$ for pre-hpx and post-hpx PCG. Even though this difference is high in percentage, this is acceptable with respect to the absolute value for clinical practice ($<3$ mmHg). The difference between the simulated and measured median of the post-hpx CO is only about 0.13 L/min ($2\%$). Nevertheless, some values attained by these outputs exceed the physiological normal ranges, in particular the computed pre-hpx and post-hpx MAP. Thus, in the following a filtering process is introduced to select only the input–output couples that respect the physiological ranges determined by clinicians.

### Sensitivity Analysis Results Using the Novel PCE-Based Approach

This section presents the GSA results obtained with the novel PCE-based methodology presented in “Classical Polynomial Chaos Expansion” section. First, the physiological filter (Table 2) is applied to the input–output couples obtained in the previous section with the full model ${\mathcal {M}}$. Second, the innovative PCE-approach is applied only on the physiological results to construct the surrogate model ${\mathcal {M}}^{\text {PCE}}$. Finally, the quality of the surrogate model ${\mathcal {M}}^{\text {PCE}}$ is verified, the new *filtered* Sobol indices results are illustrated and the so-generated virtual population is defined.

**Figure 5:**
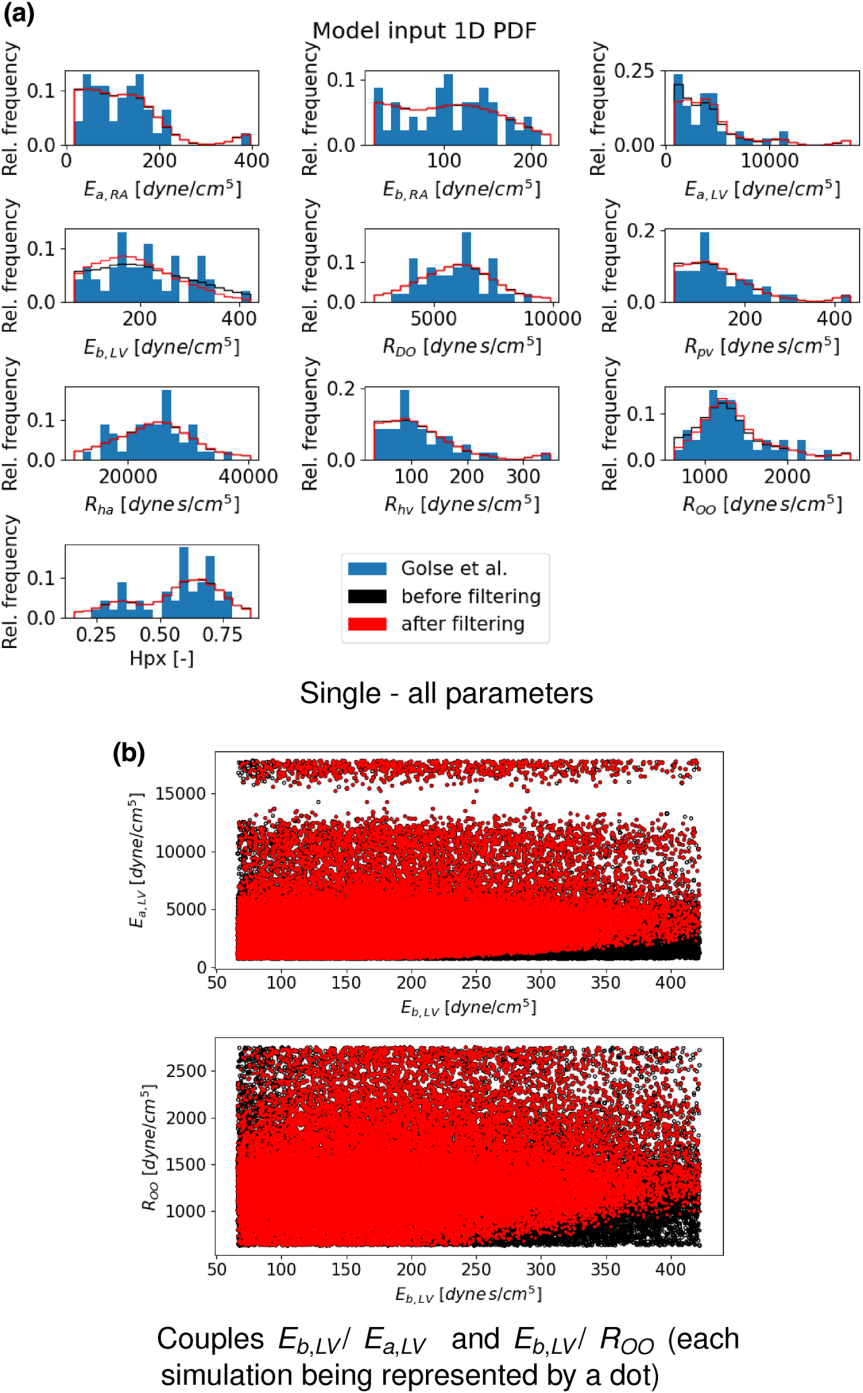
Input parameter distribution comparison before (black) and after (red) filtering, considering (**a**) one parameter at a time and the measurement distribution described in Ref. 12 (blue) or (**b**) certain couples of parameters. The *y*-axis in (**a**) displays the relative frequency, which is the ratio of the frequency of a particular event to the total frequency of that event to happen.

Figure 5 compares the input distributions before (in black) and after (in red) the filtering: the distribution shapes are very similar with the exception of $E_{{\text {a}},{\text {LV}}}$  and $E_{{\text {b}},{\text {LV}}}$. A deeper investigation, in particular considering the distribution of couples of input points, suggests that the most interesting outcomes are the relationship between $E_{{\text {b}},{\text {LV}}}$  and $E_{{\text {a}},{\text {LV}}}$, and $E_{{\text {b}},{\text {LV}}}$  and $R_{\text {OO}}$. As displayed by Fig. 5b, there are two regions that are filtered out, which therefore are not compatible with physiological predictions. This fact becomes quite relevant in the calibration step of the model when estimating these parameters from data (see “Discussion” section). Indeed this new information enables the possibility to further bound the input parameter search and to decrease the computational cost of the calibration algorithm. The results of this new strategy are discussed in “Impact on the Performances of the Calibration Step” section.

After the filtering, from a classical Sobol experiment, with $N=10^{4}$, thus $N_{\text {s}} = 1.2\times 10^{5}$ with the current setup, the remaining filtered physiological simulations are *ca*. $N_{\text {s}}^{*} = 9\times 10^{4}$.

The validity of ${\mathcal {M}}^{\text {PCE}}$ with respect to the major clinical outputs *Y* is tested against *ca*. $N_{\text {test}}^{*} = 4 \times 10^{4}$ (starting the SA study with $N_{\text {test}}=5 \times 10^{3}$) ${\mathcal {M}}$ simulations. Table 4 reports the $Q^{2}$ predictive squared correlation coefficient defined in Eq. (3), demonstrating excellent matching between ${\mathcal {M}}^{\text {PCE}}$ and ${\mathcal {M}}$.

**Table 4:** $Q^{2}$ predictive squared correlation coefficient [Eq. (3)] for each output of interest demonstrating the validity of ${\mathcal {M}}^{\text {PCE}}$

*N*	$N_{\text {test}}$	$P_{\text {pv}}$		PCG		MAP	
		Pre-hpx	Post-hpx	Pre-hpx	Post-hpx	Pre-hpx	Post-hpx
$10^{4}$	$5\times 10^{3}$	0.9983	0.9961	0.9976	0.9954	0.9982	0.9981

*N*	$N_{\text {test}}$	CO		$Q_{\text {ha}}$		$Q_{\text {pv}}$	
		Pre-hpx	Post-hpx	Pre-hpx	Post-hpx	Pre-hpx	Post-hpx
$10^{4}$	$5\times 10^{3}$	0.9986	0.9986	0.9981	0.9955	0.9983	0.9976

In order to verify that convergence is reached, several sequential simulations with increasing *N* are performed ($N = \left[ 5 \times 10^{3}, \,10^{4}, \,2 \times 10^{4},\, 4 \times 10^{4} \right]$ exploiting the simulations already completed for the results presented in “Sensitivity Analysis Results Using the Full Model” section). The error between two sets of simulations - defined by $N_{1}$ and $N_{2}$ with $N_{1}>N_{2}$, respectively—is computed as follows:

$$\begin{aligned}&{\text {err}}^{S_{1}, Y}_{N_{1},N_{2}} = \max _{j \in \left[ 1, \dots , d \right] }\left|S^{N_{1}}_{1,X_{j}} - S^{N_{2}}_{1,X_{j}}\right|,\\&{\text {err}}^{S_{\text {tot}}, Y}_{N_{1},N_{2}} = \max _{j \in \left[ 1, \dots , d \right] } \left|S^{N_{1}}_{{\text {tot}},X_{j}} - S^{N_{2}}_{{\text {tot}},X_{j}} \right|, \end{aligned}$$

where $S^{N_{i}}_{1,X_{j}}$ and $S^{N_{i}}_{{\text {tot}},X_{j}}$ are the first and the total order Sobol indices of the input variable $X_{j}$ with respect to a specific clinical output computed with $N=N_{i}$, respectively. Tables 5 and 6 detail the errors for all the major clinical hemodynamics outputs. The computed error values suggest that increasing the size from $5\times 10^{3}$ to $10^{4}$ is significantly influencing the accuracy of the Sobol indices, whereas increasing from $10^{4}$ to $2\times 10^{4}$ and $4\times 10^{4}$ is not affecting the quality of the predictions. Thus, $N=10^{4}$ is considered as the preferred choice in terms of cost-efficiency.

**Table 5:** Maximum convergence errors for the Sobol first order indices defined in Eq. (2)

	$P_{\text {pv}}$		PCG		MAP	
	Pre-hpx	Post-hpx	Pre-hpx	Post-hpx	Pre-hpx	Post-hpx
10,000–5000	0.994	0.89	0.567	0.923	0.833	0.818
20,000–10,000	0.007	0.005	0.006	0.011	0.017	0.006
40,000–20,000	0.006	0.004	0.006	0.001	0.003	0.006

	CO		$Q_{\text {ha}}$		$Q_{\text {pv}}$	
	Pre-hpx	Post-hpx	Pre-hpx	Post-hpx	Pre-hpx	Post-hpx
10,000–5000	0.984	0.923	0.635	0.91	0.916	0.808
20,000–10,000	0.006	0.006	0.006	0.011	0.007	0.006
40,000–20000	0.005	0.005	0.006	0.001	0.008	0.006

**Table 6:** Maximum convergence errors for the Sobol total order indices defined in Eq. (2)

	$P_{\text {pv}}$		PCG		MAP	
	Pre-hpx	Post-hpx	Pre-hpx	Post-hpx	Pre-hpx	Post-hpx
10,000–5000	0.984	0.867	0.581	0.916	0.807	0.797
20,000–10,000	0.007	0.006	0.006	0.011	0.017	0.006
40,000–20,000	0.006	0.004	0.006	0.001	0.004	0.007

	CO		$Q_{\text {ha}}$		$Q_{\text {pv}}$	
	Pre-hpx	Post-hpx	Pre-hpx	Post-hpx	Pre-hpx	Post-hpx
10,000–5000	0.969	0.902	0.613	0.903	0.888	0.786
20,000–10,000	0.006	0.007	0.006	0.011	0.008	0.006
40,000–20,000	0.006	0.006	0.006	0.001	0.008	0.007

Finally, to further ensure the quality of the computed results, 10 different experiments with $N=10^{4}$ are run (the random sampling every time has a different seeding, reusing again the original simulations computed for “Sensitivity Analysis Results Using the Full Model” section) to compute the variability for each Sobol index in terms of confidence intervals.

**Figure 6:**
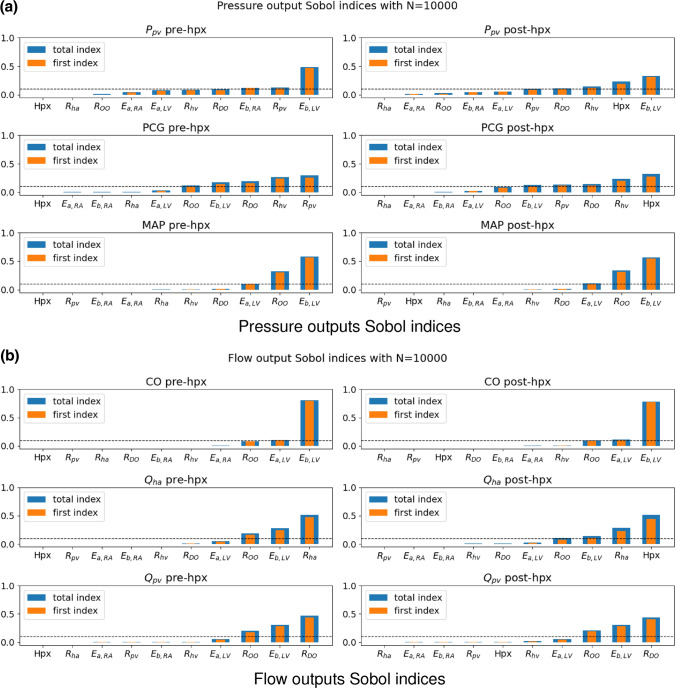
SA results after applying the physiological filter, using the PCE-based surrogate model ${\mathcal {M}}^{\text {PCE}}$ ($N=10^{4}.$)

Figure 6 illustrates the Sobol indices computed with ${\mathcal {M}}^{\text {PCE}}$. The pre-hpx results (left panels Fig. 6) indicate that:

- $E_{{\text {b}},{\text {LV}}}$  plays a significant role in all the major hemodynamics outputs: significant on $P_{\text {pv}}$, MAP, CO and $Q_{\text {pv}}$, mild on PCG and $Q_{\text {ha}}$;
- $E_{{\text {a}},{\text {RA}}}$, $E_{{\text {b}},{\text {RA}}}$  have respectively a weak and moderate influence on $P_{\text {pv}}$ and negligible influence on all the other outputs of interest;
- the effect of $R_{\text {OO}}$ is significant for all the quantities of interest, especially for MAP, except for $P_{\text {pv}}$;
- $R_{\text {ha}}$ is crucial in the determination of $Q_{\text {ha}}$ only;
- $R_{\text {DO}}$ has an impact notably on $Q_{\text {pv}}$ and moderately on $P_{\text {pv}}$ and PCG;
- variability in $R_{\text {pv}}$ and $R_{\text {hv}}$ affect the simulated values of $P_{\text {pv}}$ and PCG.

For what concerns the post-hpx predictions, right panels Fig. 6 suggests that

- $P_{\text {pv}}$ is mainly influenced by $E_{{\text {b}},{\text {LV}}}$, Hpx, $R_{\text {DO}}$, $R_{\text {pv}}$, and $R_{\text {hv}}$;
- PCG is mainly influenced by Hpx and mildly by $E_{{\text {b}},{\text {LV}}}$, $R_{\text {DO}}$, $R_{\text {pv}}$, $R_{\text {hv}}$ and $R_{\text {OO}}$;
- MAP and CO are mainly influenced by $E_{{\text {a}},{\text {LV}}}$, $E_{{\text {b}},{\text {LV}}}$  and $R_{\text {OO}}$;
- $Q_{\text {ha}}$ is mainly influenced by Hpx, $R_{\text {ha}}$, $E_{{\text {b}},{\text {LV}}}$  and $R_{\text {OO}}$;
- $Q_{\text {pv}}$ is mainly influenced by $R_{\text {DO}}$, $E_{{\text {b}},{\text {LV}}}$  and $R_{\text {OO}}$.

Similarly to the Sobol indices results employing the full model ${\mathcal {M}}$ before the filtering, the differences between the pre-hpx and post-hpx lie in the impact of Hpx. Also in this case Hpx has a negligible effect on the post-hpx $Q_{\text {pv}}$, MAP and CO with respect to $E_{{\text {a}},{\text {LV}}}$, $E_{{\text {b}},{\text {LV}}}$  and $R_{\text {OO}}$, which are the main driving parameters of the systemic blood circulation. Note that for a patient-specific simulation, the values of $Q_{\text {pv}}$, MAP and CO are influenced by the hepatectomy size Hpx, in combination with peroperative events such as blood loss or vasodilation. In the future it will be interesting to evaluate the impact of such peroperative events on these outputs and their interplay with $P_{\text {pv}}$ and PCG. This fact motivates the need of having the systemic view on the cardiovascular system despite the evident focus on the liver. Table 7 summarizes the GSA outcomes employing the surrogate model ${\mathcal {M}}_{\text {PCE}}$. Note that for every input and output couple the first index is close to the associated total index, which means that higher order interactions are negligible.

**Table 7:**
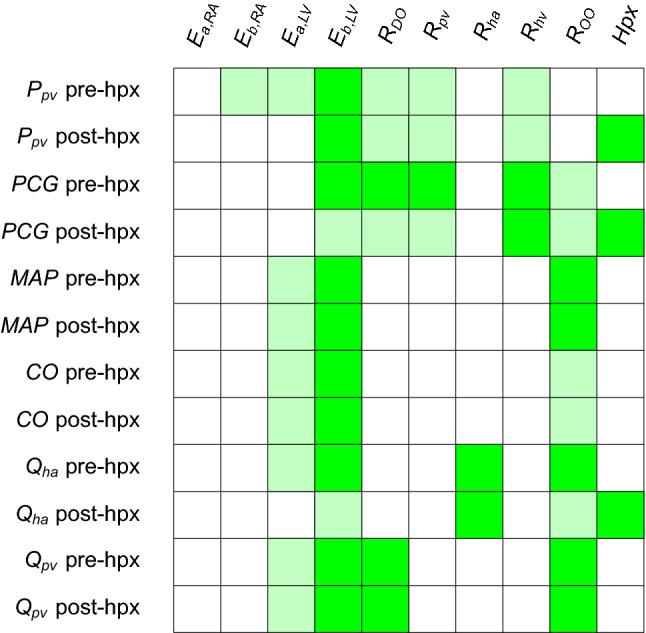
Sensitive and insensitive input parameters for each quantity of interest suggested by the GSA performed with the surrogate model ${\mathcal {M}}_{\text {PCE}}$ (see Fig. 6 for detailed representation of Sobol indices).

Legend: green = sensitive $S_{ij} \gg 0.1$, light green = fairly sensitive $S_{ij} \approx S^{\text {tot}}_{ij} \approx 0.1$, white = insensitive $S^{\text {tot}}_{ij} \approx 0$

**Comparison with clinical measurements.** Henceforth we compare the predicted output probability density functions with the clinical measurement distributions.

**Figure 7:**
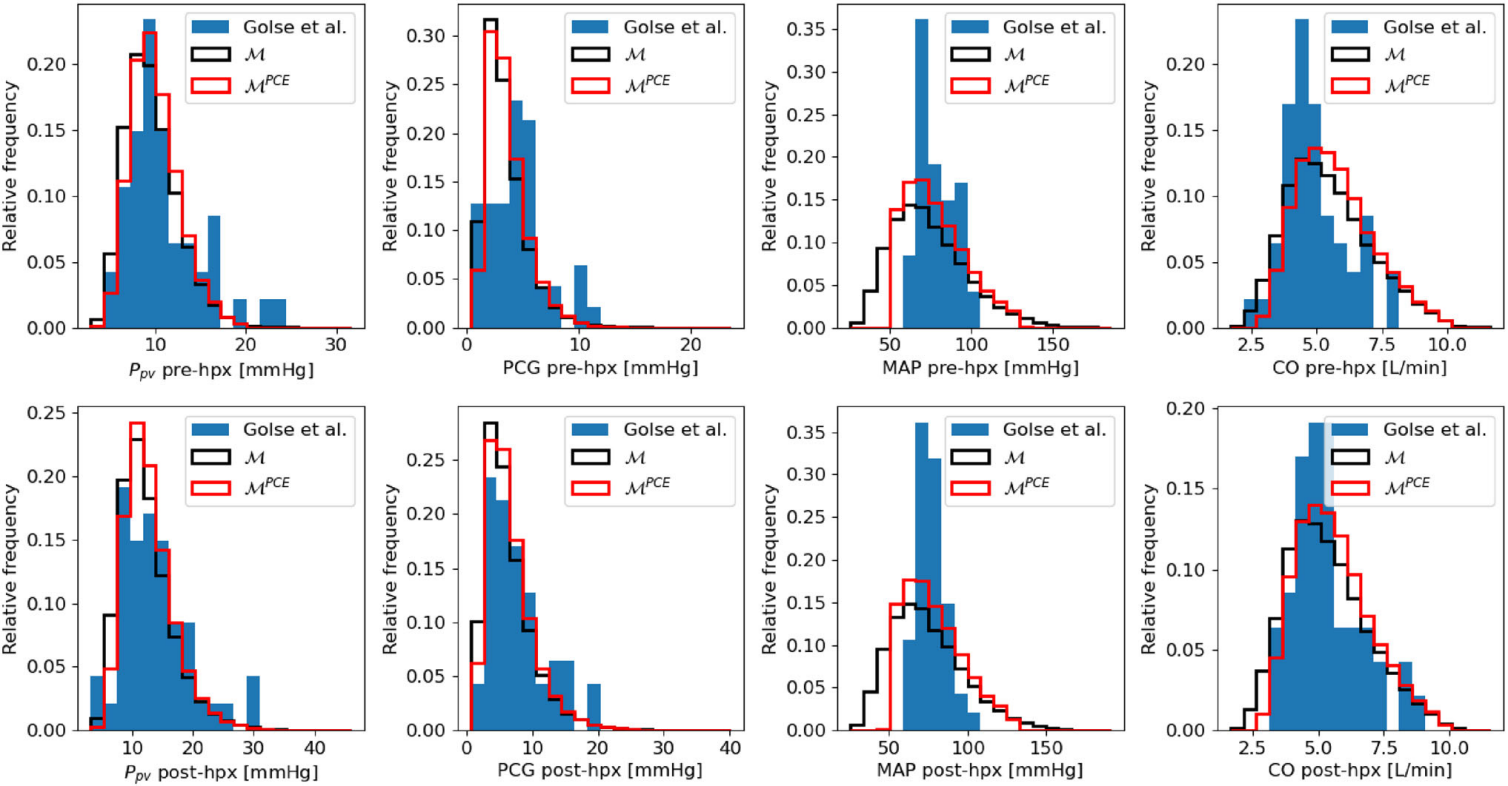
Output probability density function comparison among clinical measurements from Golse *et al*.^12^ (blue), full model ${\mathcal {M}}$ simulation results with $N=10^{4}$ (black), and PCE-based physiological surrogate model ${\mathcal {M}}^{\text {PCE}}$ simulation results with $N=10^{4}$ (red). The *y*-axis displays the relative frequency, which is the ratio of the frequency of a particular event to the total frequency of that event to happen.

The results in Fig. 7 imply an overall very good agreement between the simulations and the clinical measurements. In particular the pre-hpx predictions for $P_{\text {pv}}$ and CO share the same modal values as the relative clinical measurements. This condition is also reproduced for the post-hpx $P_{\text {pv}}$, CO and PCG. Similarly the output domain between the numerical results and the clinical measurements is comparable for pre-hpx $P_{\text {pv}}$, pre-hpx PCG, post-hpx $P_{\text {pv}}$, post-hpx PCG, and post-hpx CO. To evaluate quantitatively the accuracy of the new results, the medians of the measurement distribution (considered as baseline value) and the ones of the simulation distribution are compared. In particular for pre-hpx and post-hpx $P_{\text {pv}}$ medians the difference is below 0.4 mmHg ($3\%$), while for pre-hpx and post-hpx PCG medians the difference is 0.82 mmHg ($17\%$) and 1.22 mmHg ($20\%$), respectively. For the systemic quantity of interest, pre-hpx and post-hpx MAP medians show a difference that is below 2 mmHg (2%), while for pre-hpx and post-hpx CO medians the difference is on average 0.6 L/min (10%).

Pre-hpx simulations respect by design the physiological filter. However (i) measured pre-hpx $P_{\text {pv}}$ has higher values than simulated one (top left panel of Fig. 7), (ii) measured pre-hpx CO attains lower values than model predictions (top right panel of Fig. 7), (iii) simulated pre-hpx and post-hpx MAP have wider ranges than the measurements (mid right panels of Fig. 7), and (iv) simulated pre-hpx CO has higher values than the data distribution from Ref. 12 (top right panel of Fig. 7).

The cause is that ranges of the measurements and the ones specified in the physiological filter (Table 2) do not always match. The difference for (i) can be explained by the fact that the measurements come from a preliminary empirical study; the guidelines^10^ do not recommend to perform surgeries for such high values, which are only a small portion of the clinical cohort considered for comparison.^12^ For (ii), the anaesthesiologists believe that the few aberrant values are due to measurement error (effect of underdamping of the arterial curve). The justification of (iii) and (iv) is that the comparative measurement dataset is relatively small, hence is not completely representative of a real patient cohort.

Therefore, the ensemble of parameters and the couples input–output used in this study are a promising generated virtual population that can represent well the behavior of a real population of patients (virtual population dataset  available at 10.5281/zenodo.7034123).

### Impact on the Performances of the Calibration Step

The results presented in the previous sections support the possibility to decrease the number of calibrated parameters, speeding up the computational time to run a virtual hepatectomy. In particular, the current pipeline to solve the model includes a calibration step for the elastances in the right atrium and left ventricle. The considerations made on $E_{{\text {a}},{\text {RA}}}$  and $E_{{\text {b}},{\text {RA}}}$  suggest that during the calibration step only the left ventricle elastances can be estimated, without losing in accuracy for the post-hpx predictions. Combining this outcome with the new physiological boundaries discovered for the set of input parameters displayed in Fig. 5 in “Sensitivity Analysis Results Using the Novel PCE-Based Approach” section among $E_{{\text {a}},{\text {LV}}}$, $E_{{\text {b}},{\text {LV}}}$and $R_{\text {OO}}$, the original calibration strategy proposed in Ref. 12 is compared to this improved calibration algorithm.

Based on clinical advice, nine virtual patients are selected as representative of the diversity seen in a real patient cohort (preoperative measurements defined in Table 8). The lumped-parameter model ${\mathcal {M}}$ is then run for each synthetic patient using the two strategies, comparing the computational time and the accuracy. The latter is computed using the following formula:

4 $$ {\text {Err}}_{L^{2}} = \sqrt{\sum _{i=1}^{6} w_{i} \left( \dfrac{Y_{i}^{\text {target}} - Y_{i}^{\text {sim}}}{Y_{i}^{\text {target}}} \right) ^{2}}, $$

where $Y_{i}^{\text {target}}$ are the input data of each patient described in Table 8, $Y_{i}^{\text {sim}}$ are the computed pre-hpx values aiming to match the inputs, and $w_{i}$ are the weights reflecting the clinical confidence on each measurement. In particular, the value of the weights are the following: $w_{i} = 1$ for PCG, MAP and CO, $w_{i} = \frac{2}{3}$ for $P_{\text {pv}}$ and $w_{i} = \frac{1}{3}$ for $Q_{\text {pv}}$ and $Q_{\text {ha}}$.

**Table 8:** Input parameters for the calibration strategy comparison study

Virtual patient	MAP (mmHg)	CO (L/min)	$P_{\text {pv}}$ (mmHg)	PCG (mmHg)
*Baseline: low risk*	76	4.65	10	4
*Hypodynamics: low risk*	60	3	10	4
*Hyperdynamics: low risk*	100	7	10	4
*Baseline: high risk*	76	4.65	18	14
*Hypodynamics: high risk*	60	3	18	14
*Hyperdynamics: high risk*	100	7	18	14
*Baseline: intermediate risk*	76	4.65	15	10
*Hypodynamics: intermediate risk*	60	3	15	10
*Hyperdynamics: intermediate risk*	100	7	15	10

The values of $Q_{\text {pv}}$ and $Q_{\text {ha}}$ are automatically computed (20 and 5% of the CO, respectively, see Ref. 12). Virtual patients have different hemodynamics states and PHT risk according to EASL.^10^

The results suggest that the new algorithm outperforms the original one by almost 19%, with a small reduction in accuracy: the relative error computed with Eq. (4) is 0.03 vs. 0.02, respectively. Moreover, considering only the virtual patient cases in which the original algorithm had reached the maximum number of iterations allowed in the calibration step, the speed up of the new algorithm is on average 41% faster and with comparable precision. Note that this analysis is just a preliminary investigation on the performance of the new calibration algorithm based on a small synthetic cohort. In the future a larger database of real patients to further verify this trend will be considered.

## Discussion

This section discusses the results presented in “Results” section and their implications for future developments.

**Consequences of the physiological filter** First, as mentioned in the previous section, there is a very good agreement between the simulated and measured output probability density functions (Fig. 4), while taking into account the variability seen in the operating room. This is proving the clinical relevance of these results to define a virtual population. The comparison among the medians of the measurements and of the simulation distributions before and after the filtering suggests that the filtering outcomes are noticeably more accurate for all the outputs with the exception of the CO where the non-filtered results had already a good precision. In particular for MAP the pre-hpx and post-hpx filtered medians have an increased accuracy of 74% and 68%, respectively.

Second, the application of the physiological filter has a mild effect on the computed Sobol indices. In particular, the comparison between the computed preoperative Sobol indices before and after the filtering (left panels of Figs. 3 and 6, respectively) suggest that for all indices the effect of $E_{{\text {a}},{\text {LV}}}$  is reduced on average by 0.074 after the filter. On the other hand $E_{{\text {b}},{\text {LV}}}$  has more impact in the PCE computed Sobol indices for PCG and CO. These facts, however, are not changing the ranking of parameters influence for MAP, CO, $Q_{\text {ha}}$ and $Q_{\text {pv}}$. For $P_{\text {pv}}$ and PCG pre-hpx the ranking is slightly changing even if main considerations can be applied for Sobol indices computed with ${\mathcal {M}}$ or with ${\mathcal {M}}^{\text {PCE}}$. For $Q_{\text {pv}}$, despite the fact that the ranking is preserved, left panels of Figs. 3 and 6 indicate that the influence of $R_{\text {DO}}$ increased by 0.08 (first and total indices) after the filtering. The post-hpx discussion (right panels of Figs. 3 and 6) follows the conclusions drawn for the pre-hpx Sobol indices comparison. In particular, the results suggest that $E_{{\text {a}},{\text {LV}}}$  effect is decreased after the filtering for all considered outputs *Y* (on average for first and total indices by 0.066). The sensitive parameter ranking is preserved for MAP, CO and $Q_{\text {ha}}$, whereas it mildly changed for $P_{\text {pv}}$, PCG and $Q_{\text {pv}}$. $R_{\text {DO}}$ effect is increased after the filtering for $Q_{\text {pv}}$ by 0.08 for first and total order indices. Similarly $R_{\text {OO}}$ effect is increased after the filtering for MAP by 0.08 for first and total order indices. Finally $E_{{\text {b}},{\text {LV}}}$  effect is increased after the filtering for CO by 0.12 and 0.11 for first and total order indices, respectively.

**Comparison with literature data** Third, the Sobol indices results presented in the previous section are in agreement with respect to previous findings in literature. Central panels of Fig. 6a show that $R_{\text {DO}}$ is a sensitive parameters for PCG as also revealed by Wang *et al*.^24^ The importance of left ventricle elastance in combination with $R_{\text {OO}}$ for the MAP is consistent with the SA performed in Refs. 4, 16.

**Impact of the SA on the calibrated set from measurements** Fourth, the combination of the Sobol indices results pre-hpx and post-hpx opens to the identification of which parameters can be better calibrated in the pre-hpx in order to increase the accuracy of the post-hpx predictions. Bottom left panel of Fig. 6a and top left panel of Fig. 6b indicate that the pre-hpx value of MAP and CO can be exploited to have a good estimation of $E_{{\text {a}},{\text {LV}}}$, $E_{{\text {b}},{\text {LV}}}$and $R_{\text {OO}}$. A good calibration of these input parameters is crucial to have accurate results for all the clinical outputs considered in this analysis. If the main interest is the HA flow, the results (central panels of Fig. 6b) suggest that a precise value of pre-hpx $Q_{\text {ha}}$ would support a good calibration of the HA resistance, which is one of the main factors in the estimation of the post-hpx $Q_{\text {ha}}$. However since $R_{\text {ha}}$ has a significant impact only on post-hpx $Q_{\text {ha}}$, the patient-specific calibration of this parameter can be neglected if this clinical output is not of interest. In addition, the good calibration of $R_{\text {DO}}$ is crucial to have reliable post-hpx results for $Q_{\text {pv}}$, $P_{\text {pv}}$ and PCG. This analysis suggests that $R_{\text {DO}}$ can be efficiently estimated with an accurate measurement of the pre-hpx PV flow (left bottom panel of Fig. 6b). This fact is quite important since it motivates the need of precise measurement of $Q_{\text {pv}}$, instead of estimating it from the CO. Similarly, a precise measurement of the pre-hpx PCG is critical in the calibration of $R_{\text {pv}}$ and $R_{\text {hv}}$ and, consequently, in the computation of the post-hpx $P_{\text {pv}}$ and PCG. The complex behavior illustrated by the Sobol indices for post-hpx $P_{\text {pv}}$ and PCG (right top and central panels of Fig. 6a) is consistent with the difficulty for surgeons to foresee postoperative portal hypertension due to hepatectomy. Finally, this SA study signals a difficulty in the calibration of the right atrium elastances; indeed, they do not have a significant impact on any of the considered pre-hpx hemodynamics output (left panels of Fig. 6). However the impact of $E_{{\text {a}},{\text {RA}}}$  and $E_{{\text {b}},{\text {RA}}}$  on the post-hpx predictions is negligible (right panels of Fig. 6), thus the quality of the predictions should not be affected. A preliminary exploitation of fixing insensitive parameters has been proposed in “Impact on the Performances of the Calibration Step” section to reduce the computational cost. Other input parameters among *X* might be fixed but this analysis suggests that this would make sense only if a few quantities of interest among the current *Y* are considered.

**Limitations** This study has however some limitations. First, we selected as input parameters for the GSA the ones that were directly tuned from data in Golse *et al*.^12^ The influence of other model parameters will be investigated in future works.

Second, the data utilized to construct the empirical distributions are based on a small cohort of 47 patients, which might be not fully representative of a clinical database. Although the physiological filter partially counterbalances this, a larger cohort of patient clinical data in pre-hpx and post-hpx would support the conclusions drawn in this contribution.

Third, the simulation pipeline adopted in this study has already been shown to be improved by considering peroperative events such as blood loss or cardiac frequency changes.^12^ When their statistics will be available, these events should be taken into account to increase the quality of the model predictions. In this context the model complexity has been considered fixed. If the model requires further developments, a first stage of validation before a new GSA has to be performed; however the framework to realize such SA is proposed in this work.

Fourth, the analysis has focused only on the variability within a population. A future UQ considering uncertainties for a given subject is planned.

**Conclusion** In conclusion, from a modeling viewpoint, we have performed a GSA to define which parameters are more important for this hemodynamic model targeting surgical actions; in particular we identified which measurements can be negligible (e.g. $Q_{\text {ha}}$) and which require good accuracy (e.g. $Q_{\text {pv}}$) to provide solid predictions. From a methodological viewpoint we provided an innovative approach exploiting the features of PCE; summarizing, the surrogate model is built using only the physiological input–output couples, avoiding the need of resampling or adopting a full Monte Carlo technique which would be computationally very expensive. From these input–output couples we created a virtual population that can be then used for future studies, for instance to investigate the effect of peroperative events changes or to simulate other surgical actions such as embolization. Finally, we improved the calibration strategy using the information retrieved from the physiological filter outcome and the results of the SA, significantly decreasing the overall computational cost of simulating a virtual hepatectomy, in particular for the parameter estimation step.

**Future developments** In the future we intend to extend the GSA to other significant input modelling parameters and to investigate the uncertainties within-subjects. The virtual population generated in this study will be used to investigate the uncertainty quantification for specific patients due to preoperative measurements and per-operative events, and to employ the same approach to other types of surgical procedures for the liver, *e.g.* liver transplantation and portal embolization.

## Figures and Tables

**Figure 8:**
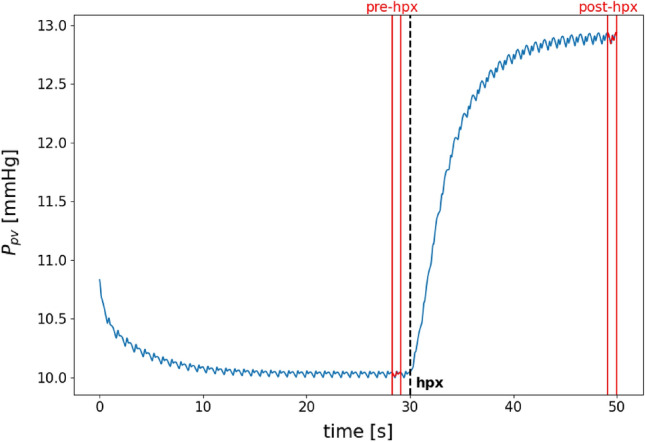
Computed $P_{\text {pv}}.$ over time

## Appendix 1: Cardiovascular Model Simulating Partial Hepatectomy

In this “Appendix”, we recall the main features of the mathematical model employed in this work that has been introduced and validated in Refs. 2, 12. This representation of the human cardiovascular system simulates the hemodynamics response to partial hepatectomy exploiting the electric analogy to fluid flow.^11^

This lumped model (Fig. 1) is composed by 6 blocks characterizing the main organs that are of clinical interest during hepatectomy. The associated nonlinear algebraic system is

5 $$ f \left( \dfrac{{\text {d}}y}{{\text {d}}t}, y, t, x \right) = 0, $$

where *y* is the state variable vector (see Table 9) and *x* is the list of all the model parameters (see Table 10). The parameter values are available in the dataset  at 10.5281/zenodo.7034123. Given the input vector *X* and quantities of interest *Y* as illustrated in “Input Parameters” and “Quantities of Interest” sections, respectively, system (5) can be rewritten as

$$ Y = {\mathcal {M}} (X), $$

where ${\mathcal {M}}$ is the notation describing the model of Fig. 1 that associates *X*, subset of *x*, and $Y = H(y,t)$ with *H* observation function which only involves a subset of y.

**Table 9:** State variables *y* of the human cardiovascular lumped-parameter model simulating partial hepatectomy introduced in Ref. 2, 12

State variable	Acronym	State variable	Acronym
Right atrium blood volume	$V_{\text {ra}}$	Right ventricle blood volume	$V_{\text {rv}}$
Left atrium blood volume	$V_{\text {la}}$	Left ventricle blood volume	$V_{\text {lv}}$
Vena cava flow	$Q_{\text {vein}}$	Tricuspid valve flow	$Q_{\text {tri}}$
Pulmonary artery flow	$Q_{{\text {a}},{\text {pul}}}$	Pulmonary vein flow	$Q_{{\text {v}},{\text {pul}}}$
Mitralic valve flow	$Q_{\text {mi}}$	Cardiac output	CO
Other organs arterial flow	$Q_{\text {a}}$	Other organs vein flow	$Q_{\text {v}}$
Mesentery flow	$Q_{\text {mes}}$	Portocaval gradient	PCG
Right atrium pressure	$P_{\text {ra}}$	Right ventricle pressure	$P_{\text {rv}}$
Left ventricle pressure	$P_{\text {lv}}$	Pulmonary arterial pressure	$P_{{\text {a}},{\text {pul}}}$
Pulmonary pressure	$P_{{\text {p}},{\text {pul}}}$	Mean arterial pressure	MAP
Other organs pressure	$P_{\text {p}}$	Mesentery pressure	$P_{{\text {p}},{\text {mes}}}$
Left portal vein flow	$Q_{{\text {pv}},{\text {L}}}$	Right portal vein flow	$Q_{{\text {pv}},{\text {R}}}$
Left hepatic artery flow	$Q_{{\text {ha}},{\text {L}}}$	Right hepatic artery flow	$Q_{{\text {ha}},{\text {R}}}$
Left hepatic vein flow	$Q_{{\text {hv}},{\text {L}}}$	Right hepatic vein flow	$Q_{{\text {hv}},{\text {R}}}$
Portal vein pressure	$P_{\text {pv}}$	Left liver pressure	${\text {Pt}}_{\text {L}}$
Right liver pressure	${\text {Pt}}_{\text {R}}$

**Table 10:** Model parameters of the human cardiovascular lumped-parameter model simulating partial hepatectomy introduced in Ref. 2, 12

Model parameter	Acronym	Model parameter	Acronym
Left liver mass	$M_{\text {L}}$	Right liver mass	$M_{\text {R}}$
Total liver mass	$M_{\text {tot}}$	% of left liver resection	${\text {Hpx}}_{\text {L}}$
% of right liver resection	${\text {Hpx}}_{\text {R}}$	% of total liver resection	Hpx
Right atrium heart elastances	$E_{{\text {a}},{\text {RA}}}$, $E_{{\text {b}},{\text {RA}}}$	Right ventricle heart elastances	$E_{{\text {a}},{\text {RV}}}$, $E_{{\text {b}},{\text {RV}}}$
Left atrium heart elastances	$E_{{\text {a}},{\text {LA}}}$, $E_{{\text {b}},{\text {LA}}}$	Left ventricle heart elastances	$E_{{\text {a}},{\text {LV}}}$, $E_{{\text {b}},{\text {LV}}}$
Right atrium rest volume	$V_{{\text {ra}},0}$	Right ventricle rest volume	$V_{{\text {rv}},0}$
Left atrium rest volume	$V_{{\text {la}},0}$	Left ventricle rest volume	$V_{{\text {lv}},0}$
Pulmonary resistance	$R_{\text {pul}}$	Pulmonary capacitance	$C_{\text {pul}}$
Other organs capacitance	$C_{\text {OO}}$	Mesentery capacitance	$C_{\text {mes}}$
Capacitance for liver mass	$C_{\text {liver}}$	Portal vein nonlinear resistance parameters	$K_{\text {pv}}, b_{\text {pv}}, c_{\text {pv}}$
Time of ventricle contraction	$T_{\text {vc}}$	Time of ventricle relaxation	$T_{\text {vr}}$
Time of atrium contraction	$T_{\text {ac}}$	Time of atrium relaxation	$T_{\text {ar}}$
Beginning of atrium contraction	$t_{\text {ac}}$	Beginning of atrium relax	$t_{\text {ar}}$
Mesentery resistance	$R_{\text {mes}}$	Portal vein resistance	$R_{\text {pv}}$
Hepatic artery resistance	$R_{\text {ha}}$	Hepatic vein resistance	$R_{\text {hv}}$
Other organs resistance	$R_{\text {OO}}$	Proximal/distal resistance ratio	$\alpha _{\text {R}}$
Heart rate	HR	Coefficient that partitions the liver pressure gradient	$\alpha _{\text {liver}}$
		between portal system and sinusoids+hepatic veins	

Right and left heart are modeled on Ref. 5. Each heart chamber—right atrium (RA), right ventricle (RV), left atrium (LA), left ventricle (LV)—is described by the following system of equations:

6a $$ \left\{ \begin{array}{ll} \frac{{\text {d}}V_{i}}{{\text {d}}t} = Q_{{\text {in}},{\text {i}}} - Q_{{\text {out}},{\text {i}}} &{} \forall i \in \left\{ {\text {RA}}, {\text {RV}}, {\text {LA}}, {\text {LV}} \right\} \\ P_{i} = E_{i}(t) (V_{i} - V_{0,i}) &{} \forall i \in \left\{ {\text {RA}}, {\text {RV}}, {\text {LA}}, {\text {LV}} \right\} \\ Q_{{\text {out}},{\text {i}}} = G_{i}(\Delta P) \; \Delta P &{} \forall i \in \left\{ {\text {RA}}, {\text {RV}}, {\text {LA}}, {\text {LV}} \right\} , \end{array}\right. $$

where $V_{i}$ and $V_{0,i}$ are the volume and unloaded volume of the heart chamber *i*, respectively; $Q_{{\text {in}},{\text {i}}}$ and $Q_{{\text {in}},{\text {i}}}$ are the incoming and outgoing flows of the heart chamber *i*, respectively; $P_{i}$ is the heart chamber pressure; $\Delta P$ is the pressure drop across the valve; $G_{i}(\Delta P)$ is the valve conductance of heart chamber *i* dependent on $\Delta P$; $E_{i}$ is the elastance function, defined by

6b $$ E_{i}(t) = E_{{\text {a}},{\text {i}}} e_{i}(t) + E_{{\text {b}},{\text {i}}} \quad \forall i \in \left\{ {\text {RA}}, {\text {RV}}, {\text {LA}}, {\text {LV}} \right\} . $$

$E_{{\text {a}},{\text {i}}}$ and $E_{{\text {b}},{\text {i}}}$ are the amplitude and the baseline elastances, respectively, while *e*(*t*) is a normalized time-varying function of the elastance:

6c $$\begin{aligned}&\forall i \in \left\{ {\text {RV}}, {\text {LV}} \right\} \quad e_{i}(t) = \left\{ \begin{array}{ll} \frac{1}{2} \left[ 1- \cos \left( \pi \frac{t}{T_{\text {vc}}}\right) \right] &{} 0 \le t \le T_{\text {vc}}, \\ \frac{1}{2} \left[ 1 + \cos \left( \pi \frac{t-T_{\text {vc}}}{T_{\text {vr}}}\right) \right] &{} T_{\text {vc}} \le t \le T_{\text {vr}} + T_{\text {vc}}, \\ 0 &{} T_{\text {vc}} + T_{\text {vr}} \le t \le T_{\text {cc}}, \end{array}\right. \end{aligned}$$

6d $$\begin{aligned}&\forall i \in \left\{ {\text {RA}}, {\text {LA}} \right\} \quad e_{i}(t) = \left\{ \begin{array}{ll} \frac{1}{2} \left[ 1 + \cos \left( \pi \frac{t+T_{\text {cc}}-t_{\text {ar}}}{T_{\text {ar}}}\right) \right] &{} 0 \le t \le t_{\text {ar}} + T_{\text {ar}} - T_{\text {cc}}, \\ 0 &{} t_{\text {ar}} + T_{\text {ar}} - T_{\text {cc}} \le t \le t_{\text {ac}}, \\ \frac{1}{2} \left[ 1- \cos \left( \pi \frac{t-t_{\text {ac}}}{T_{\text {ac}}}\right) \right] &{} t_{\text {ac}} \le t \le t_{\text {ac}} + T_{\text {ac}}, \\ \frac{1}{2} \left[ 1 + \cos \left( \pi \frac{t-t_{\text {ar}}}{T_{\text {ar}}}\right) \right] &{} t_{\text {ac}} + T_{\text {ac}} \le t \le T_{\text {cc}}, \end{array}\right. \end{aligned}$$

where $T_{\text {cc}}$ is the duration of the cardiac cycle. $T_{\text {vc}}$, $T_{\text {ac}}$, $T_{\text {vr}}$, and $T_{\text {ar}}$ are the ventricular and atrial contractions and relaxations duration, respectively; $t_{\text {ac}}$ and $t_{\text {ar}}$ are the starting times of contraction and relaxation, respectively. These time intervals are scaling as functions of the heart rate which value is described in Table 2 of Ref. 2.

The liver is described by two blocks (left liver l, right liver r) in parallel both perfused by venous blood to detoxify through the portal vein (PV) and arterial blood *via* the hepatic artery (HA). The resistances $R_{{\text {pv}},{\text {r}}}$, $R_{{\text {pv}},{\text {l}}}$, $R_{{\text {ha}},{\text {r}}}$ and $R_{{\text {ha}},{\text {l}}}$ are inversely proportional to the hemiliver mass, whereas capacitances $C_{{\text {liver}},{\text {r}}}$ and $C_{{\text {liver}},{\text {l}}}$ are directly proportional to the hemiliver mass.

Lungs (LL), digestive organs (DO), and other organs (OO) are characterized by RCR three-element Windkessel model.

Given a choice of parameters as input, the coupled algebraic–differential system, representing the lumped-parameter model ${\mathcal {M}}$ described above, is solved for pressures and flows of the system over time. In particular, the model is solved for several cardiac cycles before simulating the partial hepatectomy, which is performed by decreasing the mass of the left and/or right liver. This modification has a direct impact on the liver resistance and capacitance values, thus influencing the system hemodynamics. See the standard solving pipeline in Ref. 12 for more details.

## Appendix 2: Parameter Estimation

The distributions of the input parameters specified in “Input Parameters” section come from the study of Golse *et al*.^12^ The authors employed the mathematical model presented in “Human Cardiovascular Lumped-Parameter Model” section to perform a validation study on a cohort of 47 patients. The preoperative measurements collected, as annotated in the paper, were

- PV pressure $P_{\text {pv}}$, measured by a transducer connected to a needle inserted in the portal vein;
- portocaval gradient PCG, which is the pressure difference of $P_{\text {pv}}$ and the inferior vena cava pressure, measured by a transducer connected to a needle inserted into the vessel;
- systemic arterial pressure MAP, measured using an arterial catheter;
- cardiac output CO, estimated from the thermodilution technique or pulse contour analysis;
- blood flow in the HA ($Q_{\text {ha}}$) and in the PV ($Q_{\text {pv}}$) adopting the approximation from the CO (5 and $20\%$, respectively), and also measurable with an ultrasound flowmeter.

Starting from these preoperative measurements of the cohort presented in Ref. 12, the input parameters were computed in the following way:

7a $$\begin{aligned} R_{\text {pv}}&= \dfrac{P_{\text {pv}} - P_{\text {liver}}}{Q_{\text {pv}}}, \end{aligned}$$

7b $$\begin{aligned} R_{\text {ha}}&= \dfrac{{\text {MAP}} - P_{\text {liver}}}{Q_{\text {ha}}}, \end{aligned}$$

7c $$\begin{aligned} R_{\text {hv}}&= \dfrac{P_{\text {liver}}-P_{\text {vc}}}{Q_{\text {pv}} + Q_{\text {ha}}}, \end{aligned}$$

7d $$\begin{aligned} R_{\text {DO}}&= \dfrac{{\text {MAP}} - P_{\text {pv}}}{Q_{\text {pv}}}, \end{aligned}$$

7e $$\begin{aligned} R_{\text {OO}}&= \dfrac{{\text {MAP}}-P_{\text {vc}}}{{\text {CO}} - Q_{\text {pv}} - Q_{\text {ha}}}, \end{aligned}$$

where $P_{\text {vc}} = P_{\text {pv}} -{\text {PCG}}$ is the pressure in the inferior vena cava and $P_{\text {liver}} = P_{\text {pv}} - \alpha _{\text {liver}} \, {\text {PCG}}$ is the estimated pressure within the liver with $\alpha _{\text {liver}}=0.5$ considered as constant model parameter (we refer to Ref. 2 for more details). The heart elastances in the right atrium and left ventricle are estimated using an optimization algorithm (covariance matrix adaptation evolution strategy) in order that the model predictions on the main hemodynamics output ($P_{\text {pv}}$, PCG, MAP, CO, $Q_{\text {pv}}$, $Q_{\text {ha}}$) match the clinical measures for each patient. The remaining heart elastances (right ventricle and left atrium) are changed proportionally to $E_{{\text {a}},{\text {RA}}}$, $E_{{\text {b}},{\text {RA}}}$, $E_{{\text {a}},{\text {LV}}}$  and $E_{{\text {b}},{\text {LV}}}$^12^.

## Appendix 3: Computation of Pre-hpx and Post-hpx Mean Values

This Appendix explains briefly how the scalar pre-hpx and post-hpx value of a specific hemodynamic variable is computed starting from its time-dependent evolution. The strategy is to compute the mean of this variable over a cardiac cycle just before the virtual hepatectomy (pre-hpx) or just before the end of the simulation (post-hpx). As example Fig. 8 shows the temporal evolution during the simulation of the simulated portal vein pressure (blue line). The virtual hepatectomy occurs at $T=30$ s, marked with a black vertical dashed line. The pre-hpx and post-hpx cardiac cycles selected for the computation of the mean are highlighted between two red vertical lines in the figure.

The virtual hepatectomy is performed after a certain number of cardiac cycles to let the system reach a periodic state, then the pre-hpx value is computed. For the post-hpx value, in a similar way, the computation of the mean of the variable over a cardiac cycle waits till the system has reached the new periodic state.
